# Hereditary spherocytosis with successful splenectomy in a pregnant black South African lady: a case report

**DOI:** 10.11604/pamj.2019.33.288.18873

**Published:** 2019-08-07

**Authors:** Khaled Elmezughi, Chukwuma Ekpebegh

**Affiliations:** 1 Department of Internal Medicine, Walter Sisulu University, Mthatha, South Africa

**Keywords:** Haemolytic anemia, hereditary spherocytosis, massive splenomegaly, splenectomy, pregnancy, South Africa

## Abstract

Hereditary spherocytosis is a rare cause of chronic haemolytic anaemia. It is rarer in the black population with extremely few cases reported. Initial assessment of a patient with suspected disease should include documenting clinical features of chronic haemolysis and a family history. Its management in pregnancy is challenging and needs a multidisciplinary team. We report on a black South African woman with hereditary spherocytosis and massive splenomegaly presenting with severe haemolytic anaemia at 23^rd^ week of pregnancy. She had a successful splenectomy.

## Introduction

Hereditary spherocytosis (HS) is the most common inherited red cell membrane disorder causing haemolytic anaemia [1]. It is the most common inherited haemolytic anaemia in Caucasians with a population incidence of 1:2500-5000 [1-4]. Hereditary spherocytosis has also been reported in other racial groups [2-4]. There are, however, rare reports in the black population. It is commonly inherited in autosomal dominant pattern; autosomal recessive inheritance is also reported, and some genetic studies have revealed that an unexpectedly large number of nondominant cases are due to de novo mutations in the HS genes [1]. Deficiency in red blood cell membrane proteins, ankyrin and spectrin, causes HS [1, 2]. These are major proteins that act as cytoskeleton of the red blood cell and as a result of defective membrane, red blood cells become spherically shaped; the resulting spherocytes become trapped in the spleen as they course through the sinuses, and the red cells are engulfed by macrophages. It is particularly important to exclude stomatocytosis where splenectomy is contraindicated because of the thrombotic risk [2, 3]. Hereditary spherocytosis may be mild, moderate or severe. Mild disease needs no treatment. Moderate disease is treated with folic acid at daily dosage of 5mg. Severe disease requires close haematological supervision and are likely to benefit from splenectomy, which should be performed after the age of 6 years with appropriate counselling on the subsequent risk of infection post-splenectomy [2, 3]. We report a case of a black South African lady, primigravida, with severe HS and massive splenomegaly diagnosed during pregnancy. She had an uneventful pregnancy following successful splenectomy that was performed in the second trimester of pregnancy.

## Patient and observation

A 22-year old lady from the rural eastern Cape Province of South Africa, 23 weeks pregnant was referred to the Nelson Mandela Academic Hospital (NMAH) from a base Hospital with history of severe symptomatic anaemia (Hb 5.6g/L) and massive splenomegaly. On further enquiry she reported intermittent yellowish discoloration of her eyes for the past 4 years. She also gave a history of previous blood transfusions on multiple occasions over the past 4 years. She had not been investigated for the recurring episodes of anaemia and jaundice. Family history revealed that her late father had similar episodes of recurring anaemia and jaundice but with no established diagnosis. She had never travelled to any malaria zones. Examination revealed a young lady, who was pale, jaundiced, but with no lymphadenopathy and she had a massive splenomegaly with no hepatomegaly or ascites. The rest of the examination was unremarkable. Laboratory results showed severe normocytic, normochromic anaemia, with marked reticulocytosis (reticulocyte count of 10.97%, Reticulocyte Production Index (RPI) of 2.6), low haptoglobin level, unconjugated hyperbilirubinemia with positive urobilinogen on urine dipsticks. Her blood smears confirmed the presence of spherocytes. Coombs test was negative. Serum compliments C3 and C4 level were normal. Serum anti-nuclear antibody was negative. Glucose-6-Phosphate Dehydrogenase (G6PD) assay was normal. Serum iron studies and haemoglobin electrophoresis were normal. The rest of the laboratory tests at presentation are shown on Table 1. Osmotic Fragility test (OFT) was not done.

**Table 1:** laboratory results at presentation and 8 week post splenectomy

	At presentation	8 weeks post splenectomy	Normal range
Hb (g/L)	5.6	12.6	12-15
WBC [x10^9^/L]	6.6	8.5	3.9-12.6
Platelets [x10^9^ /L]	202	478	186-454
Haematocrit [g/dL]	0.166	0.37	0.36-0.46
MCV [fL]	88.5	89.6	78.9-98.5
MCH [pg]	27.8	29.7	26.1-33.5
MCHC [g/dL]	33.4	33.2	32.7-34.9
Distribution Width [%]	25.9	18.0	12.4-17.3
Haptoglobin [g/L]	0.01	Not repeated	0.3-2.0
Retic count [%]	10.97	2.45	0.5-2.0
Na+ [mmol/L]	136	141	136-145
K+ [mmol/L]	3.1	4.4	3.5-5.1
Cl− [mmol/L]	101	104	98-107
Urea [mmol/L]	4.6	2.9	1.4-5.4
Creatinine [umol/L]	53	47	49-90
Total protein [g/L]	68	63	57-80
Albumin [g/L]	41	36	29- 42
Bilirubin [umol/L]	82	4	5-21
Direct bilirubin [umol/L]	9	2	0-3
ALT [U/L]	14	10	5-20
GGT [U/L]	14	18	4-24
ALP [U/L]	42	116	42-98

**ALT**, alanine aminotransferase; **AST**, aspartate aminotransferase; **GGT**, gamma-glutamyl transferase, **ALP**, alkaline phosphatase

Based on the clinical and laboratory investigations which are in keeping with chronic haemolysis (recurring anaemia and jaundice), spherocytosis with negative Coombs test, our patient was diagnosed as having severe haemolytic anaemia due to hereditary spherocytosis in pregnancy. She was continued on folic acid 5mg daily. A decision on splenectomy was taken after consultation with the obstetrics team as she was likely to require further red cell transfusions in the course of pregnancy. She was transfused with five units of red cells on 2 separate occasions and vaccinated against pneumococcus, meningococcus and H influenza. The immediate postoperative period was uneventful. The histopathology report confirmed a spleen size of 1028g, measuring 20X15X5.5cm (splenic tissue with congested red pulp noted and no granulomas, pathogens or evidence of malignancy). She was counselled on the importance of regular follow-up and advised to inform her family members on screening for hereditary spherocytosis. She was reviewed 6 weeks splenectomy at our Outpatient Clinic and was found to be in a good general condition without anaemia and with significant regression of jaundice. The results of laboratory tests repeated 8 weeks after splenectomy are shown in Table 1. She is currently being followed up by the obstetrics unit and is expectant of her first baby.

## Discussion

The presence of chronic recurring symptomatic anaemia, jaundice, laboratory features of haemolysis (raised serum unconjugated bilirubin, reticulocytosis, low serum haptoglobin and increased urobilinogen in urine), spherocytes on peripheral blood film, and a similar history in her father are in keeping with HS. This is particularly so, given the negative Coombs test that argues against auto-immune haemolytic anaemia as a cause of spherocytosis. The clinical severity of HS varies from symptom free carrier to severe haemolysis [2, 3]. Mild HS can be difficult to identify because individuals may have normal haemoglobin and bilirubin levels. Severe HS is defined as Hb <6g/dL, reticulocyte count >10%, bilirubin >51.3umol/L [2, 3]. Our patient, based on the levels of haemoglobin, reticulocyte count and serum bilirubin, meets the criteria for severe HS (Table 1). It is therefore not surprising that our patient had several previous blood transfusions. The demands for blood transfusions in our pregnant primigravida patient may be further worsened by traditional contributory factors for anaemia in pregnancy like haemodilution and increased requirements for folate and iron.

Our patient as with most persons with HS had splenomegaly. Manifestations of hypersplenism such as severe haemolysis rather than splenomegaly per se is an indication for splenectomy [2-4]. It is noteworthy, that serum levels of unconjugated bilirubin and reticulocyte count dramatically dropped to normal levels in our patient following splenectomy. Furthermore, her haemoglobin levels remained normal at review 6 weeks post splenectomy underscoring the benefit of splenectomy in our patient. Reports of HS in pregnancy have ranged from none requirements of splenectomies to splenectomies in pregnancy [5]. Splenectomy is effective in reducing haemolysis, leading to a significant prolongation of the red cell lifespan, although not necessarily to normal [2-4]. Patients with severe disease who are not completely cured by splenectomy have remarkable amelioration in symptoms following removal of the spleen [2, 3]. The rare splenectomy failure is usually associated with an accessory spleen [6]. The second trimester is the optimal time to perform surgery as in our patient due to increased risk of teratogenicity in the first trimester from exposure to anaesthetic agents and the technical difficulty due to obstruction of the surgical field by the gravid uterus as well as the increased risk of preterm labour in the third trimester. There is a risk of pregnancy associated life threatening aplastic crisis, haemolytic crisis and thrombosis because of life threatening sepsis from encapsulated organisms. Splenectomy also reduces the occurrence of leg ulcerations and gall stones [2-4]. Our patient however, neither had lower limb ulcerations or gall stones on abdominal ultrasound imaging. Post splenectomy antibiotic prophylaxis and immunization against pneumococcus, meningococcus and haemophilus influenza, like what our patient has received, has been shown to reduce, and not eliminate the risk of post splenectomy sepsis [2]. The limitations in our case report include the non-performance of OFT and glycerol lysis tests. These tests are not available in our setting.

## Conclusion

Hereditary spherocytosis is a rare condition and rarer in black ethnicity. High index of suspicion is necessary for its diagnosis. Our case is a black African lady, primigravida, in the second trimester who had a successful splenectomy.

## Competing interests

The authors declare no competing interests.

## References

[1] Perrotta S, Gallagher PG, Mohandas N (2008). Hereditary spherocytosis. Lancet.

[2] Bolton-Maggs PH1, Stevens RF, Dodd NJ, Lamont G, Tittensor P, King MJ (2004). Guidelines for the diagnosis and management of hereditary spherocytosis. Br J Haematol.

[3] Bolton-Maggs PH, Langer JC, Iolascon A, Tittensor P, King MJ (2012). General Haematology Task Force of the British Committee for Standards in Haematology. Guidelines for the diagnosis and management of hereditary spherocytosis-2011 update. Br J Haematol.

[4] Da Costa L, Galimand J, Fenneteau O, Mohandas N (2013). Hereditary spherocytosis, elliptocytosis, and other red cell membrane disorders. Blood Rev.

[5] Moore A, Sherman MM, Strongin MJ (1976). Hereditary spherocytosis with hemolytic crisis during pregnancy, treatment by Splenectomy. Obstet Gynecol.

[6] Mackenzie AE, Elliot DH, Eastcott HH, Hughes-Jones N, Barkhan P, Mollison PL (1962). Relapse in hereditary spherocytosis with proven splenunculus. Lancet.

